# Oleogels—Innovative Technological Solution for the Nutritional Improvement of Meat Products

**DOI:** 10.3390/foods12010131

**Published:** 2022-12-27

**Authors:** Simona Perța-Crișan, Claudiu-Ștefan Ursachi, Bianca-Denisa Chereji, Florentina-Daniela Munteanu

**Affiliations:** Faculty of Food Engineering, Tourism and Environmental Protection, “Aurel Vlaicu” University of Arad, 2–4 Elena Drăgoi Str., 310330 Arad, Romania

**Keywords:** oleogels, oleogelators, fats substitutes, innovative foods, meat products, nutritional profile

## Abstract

Food products contain important quantities of fats, which include saturated and/or unsaturated fatty acids. Because of a proven relationship between saturated fat consumption and the appearance of several diseases, an actual trend is to eliminate them from foodstuffs by finding solutions for integrating other healthier fats with high stability and solid-like structure. Polyunsaturated vegetable oils are healthier for the human diet, but their liquid consistency can lead to a weak texture or oil drain if directly introduced into foods during technological processes. Lately, the use of oleogels that are obtained through the solidification of liquid oils by using edible oleogelators, showed encouraging results as fat replacers in several types of foods. In particular, for meat products, studies regarding successful oleogel integration in burgers, meat batters, pâtés, frankfurters, fermented and bologna sausages have been noted, in order to improve their nutritional profile and make them healthier by substituting for animal fats. The present review aims to summarize the newest trends regarding the use of oleogels in meat products. However, further research on the compatibility between different oil-oleogelator formulations and meat product components is needed, as it is extremely important to obtain appropriate compositions with adequate behavior under the processing conditions.

## 1. Introduction

The consumption of saturated and trans-fats has been shown to have negative effects on human health, which have been highlighted in many studies and articles published in the popular press and scientific journals over the last decades [1,2,3,4]. 

Due to this unfavorable publicity, food producers are under pressure to seek other techniques for structuring lipid-based food items without considerable quantities of saturated and trans-fats. As the consumption of trans-fatty acids and saturated fatty acids have been linked to health issues, the Food and Agriculture Organization (FAO) recommends reducing the consumption of saturated fatty acids [5]. 

Recently, several studies have paid attention to the connection between saturated fat intake and the risks for cardiovascular disease, diabetes, cancer or mortality [6,7,8,9,10,11].

Siri-Tarino et al. concluded in 2010 that there is no sufficient evidence for linking saturated fat intake with cardiovascular disease [11]. Later, the Schwab research group showed that the replacement of saturated fats with polyunsaturated fatty acids (PUFA) has a beneficial role in the reduction of cardiovascular disease, especially in men [10], while Chowdary et al. [6] concluded that there is not sufficient evidence to sustain the high consumption of PUFA to the detriment of total saturated fats.

Another interesting study shows that the mortality causes associated with cardiovascular disease, coronary heart disease, ischemic stroke and diabetes are not directly linked to saturated fat intake but rather to industrial trans fats intake [7].

In a paper published in 2015, Hooper et al. showed that it might be possible to reduce cardiovascular events if the total intake of saturated fat is reduced [8], and later, in 2020, they published an update to their previous review and concluded that the reduction of saturated fat intake for at least two years has a beneficial effect on the reduction of combined cardiovascular events [9]. Another comprehensive review shows that the use of PUFA might have a beneficial effect on health, such as antiaging, antioxidant, antihypertensive, anticancer, antidepression, and anti-arthritis effects, but further studies and a multi-analysis are highly demanded to avoid any potential biases [12].

Vegetable fats and oils play a vital part in the human diet and usual food preparation practices. The characteristics of the various fat phases span a broad spectrum, fitting the requirements of several applications. Formulation of food products takes into account the liquid or solid lipid phase, where the liquid corresponds to vegetable oils [13]. 

The selection of raw materials plays an important role in the formulation of food products and this is not only based on economic considerations. Obviously, sourcing based on price and availability, which in this context includes volume and origin flexibility, is vital. Chemical stability and nutritional value are dependent on the content of fatty acids in the fat phase. Formulation of food products which use oils is helpful not only for human health but also from environmental perspectives [14].

Triglycerides are the primary components of fats and oils. Triglycerides may comprise a variety of fatty acids, including saturated, monounsaturated and polyunsaturated forms. Most of the time, food contains a combination of these various triglycerides [15]. 

Low-fat diets have been heavily pushed in the past since it was believed that fats may harm the health of consumers. Recent research has demonstrated that only trans and saturated fats are related to an increased risk of cardiovascular disease. However, both saturated and trans-fatty acids have an important role in the food industry [16,17]. 

This is because these acids are responsible for the taste, palatability and texture of the meals they are used in. In addition, the triacylglycerols may arrange themselves into a colloidal multi-level network, which can turn fats into solid or solid-like materials and give foods their structure [18].

Since recent legislation has prohibited the use of manufactured trans-fats and since numerous organizations have proposed limiting the amount of saturated fats in meals, there has been an increasing interest in functional lipids, as well as nutritional improvements of foods that include lipids [19,20,21,22]. 

The World Health Organization (WHO) recommends a dietary fat consumption between 1–30% of the total caloric intake in the form of saturated, trans and total fats, respectively. Regarding the problem of trans fats, the Food and Drug Administration (FDA) announced its conclusive finding that partially hydrogenated oils, the primary source of artificial trans-fats in processed foods, are not generally recognized as safe [23,24]. 

The WHO launched a campaign to analyze the dietary sources of industrially-produced trans-fats, encouraging and promoting their replacement with healthier fats and oils, adopting regulatory laws and actions to eliminate industrially-produced trans-fats, assessing and checking the trans-fat content in the food supply, observing consumers’ preferences, raising their awareness of the implications of fats on health, and enforcing compliance with policies and regulations [24]. 

The European Food Safety Authority (EFSA) approved a report for consumers that focuses on goals and recommendations regarding the intake of trans-fats and health issues. The report shows the direct relationship between the increased dose of fatty acids and the risk of cardiovascular heart disease. Therefore, the European Commission (EU) restricted the use of trans-fats to 2 g per 100 g of total fat. This limitation does not apply to trans-fats that exist naturally in the fat of animal origin, as is mentioned in the relatively recent Regulation No. 649/24 April 2019 [25].

The present review focuses on the latest research on replacing animal fats in meat products with healthy, fat-rich, vegetable oil-based, nutritionally balanced ingredients with good sensorial and textural characteristics so that consumer acceptability is not significantly influenced. Therefore, the use of oleogels for this purpose is seen as an innovative solution for the formulation of nutritionally improved meat products. 

## 2. Oleogels—Novel Suitable Substitutes for Animal Fats from Foods

### 2.1. Definition and Description

Oleogels or structured oils can be defined as micro-structured three-dimensional systems obtained by the solidification of edible liquid oils, in the presence of so-called oleogelator(s) [26]. They are considered to be semi-solid materials in which the liquid oil fraction is immobilized in a network of structuring molecules [27].

**Figure 1:**
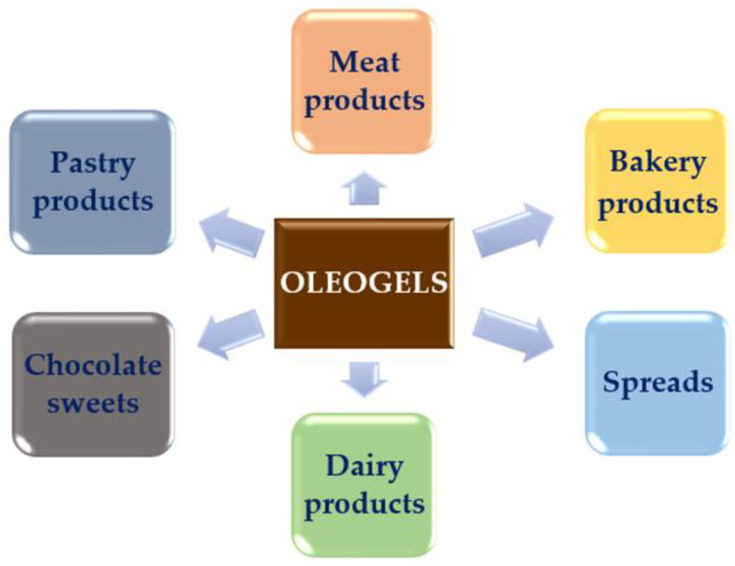
Research fields for applicability of oleogels in foods.

Oleogels are a healthier alternative to saturated fats and trans-fats, and they are finding significant uses in many different sectors of the food industry [26]. As shown in Figure 1, these areas include the baking industry, meat industry, confectionery industry, and dairy industry, amongst others. The baking industry has benefited the most from the formulation of oleogels since these can be used as replacers for shortenings and spreads which then contain no trans-fats and a decreased quantity of saturated fats.

Therefore, it is extremely important to investigate different methods of changing liquid oil into solid fats that include no trans-fatty acids and just a small amount of saturated acid. The innovative and potentially fruitful alternative technique, known as oleogelation, has lately attracted significant interest [28].

Oleogel is a thermo-reversible lipid combination with high viscoelastic properties that may exist in a semi-solid state. The oleogelators are combined with a lipophilic liquid (often vegetable oil) to form the emulsion. Liquid oil may be captured by oleogelators, which then crystallize or self-assemble into different shapes. This results in a three-dimensional network structure and gelatinization of the entire system, which stops lipophilic liquid from flowing through the system [29].

Oleogel has the ability not only to preserve the solid features of foods, but also to provide the health benefits associated with low levels of saturated fatty acids. A suitable oleogel for edible purposes should have changeable physical and chemical characteristics to fulfil other intended functions, such as controlled release and excellent bioavailability [30]. 

Researchers consider that oleogels have a wide range of applications and a significant unexploited potential. Their use allows the obtaining of products with acceptable technological properties and may combat oil leaks in a wide range of different foods. Other important applications for oleogels include their use as carriers for lipophilic bioactive substances [31,32].

Several studies show that oleogels boost lipid-soluble molecule bioavailability. For example, the incorporation of β-carotene in oleogels benefits from increased bioavailability and retardation of lipid oxidation. Moreover, it is possible to control the release of this nutraceutical in the bloodstream [33,34]. 

Another example is the release of curcumin, a water-insoluble nutraceutical molecule with several health benefits, which is included in oleogels. For the oleogel preparation, medium-chain triacylglycerols (canola, coconut, or maize oils) were used, in which curcuminoid was dissolved under heating. Thereafter, mono-stearin, as an artificial trans fat oleogelator, was added. It was shown that the bioavailability was greater for the curcuminoid-oleogel than for the curcuminoid powder distributed in the water [35].

Besides improving nutritional value, the incorporation of oleogels into food products can be attractive in terms of stability and shelf life. Due to their three-dimensional structures, oleogels constitute a good matrix for the delivery of bioactive molecules [36], such as antioxidants [37], probiotics [38] or antimicrobial compounds [39,40,41]. However, this last area is not yet widely studied and existing reports generally focus on the use of fat-soluble compounds, due to the lipophilic nature of oleogels [42]. 

Currently, there are very few studies on the microbiological aspects of oleogels. Due to their composition, high oil content and low water activity, oleogels are not a favorable environment for microorganisms. Furthermore, several studies have shown that beeswax, a commonly used oleogelator, has inhibitory effects on a variety of microbes such as *Salmonella enterica*, *Staphylococcus aureus*, *Candida albicans* or *Aspergillus niger* [43,44,45].

According to Pintado et al. (2020) [46], the strategy of reducing and replacing animal fat in dry fermented sausages with an oleogel obtained by structuring a mixture of olive and chia oils with 10% beeswax resulted in microbiologically safe products. Another study showed that it is possible to produce microbiologically safe fermented sausages by partially substituting the pork backfat with olive oil/monoglycerides oleogel [47]. 

Bei et al. (2015) [39] developed a stable stearic acid and peanut oil oleogel-based emulsion supplemented with D-limonene and nisin, two natural compounds with antimicrobial activity. The results indicate that the addition of the two antimicrobial agents showed remarkable inhibitory effects against all target microorganisms (*Escherichia coli*, *Bacillus subtilis* and *S. aureus*). Moreover, the addition of the organogel-nano-emulsion with D-limonene and nisin as food preservatives in milk samples also demonstrated notable antimicrobial activity.

In another study, modified whey protein isolate was used to prepare emulsion-based oleogels with thyme essential oil and coconut oil. The resulting product exhibited a regular, ordered, porous network structure with good antimicrobial activities against *Escherichia coli* [40].

Yadav et al. (2017) developed an oleogel from rice bran oil and candelilla wax supplemented with ciprofloxacin hydrochloride, a well-known antibiotic [41]. Antimicrobial studies showed that this compound maintained its antimicrobial properties and the oleogel was able to resist the growth of *Escherichia coli*.

In recent years, the possibility of using oleogels as an alternative method of structuring oils has been recognized and this possibility has been deeply examined by using a variety of gelator-edible oil systems. These unique oil-based oleogels contain a lipidic continuous phase and display the physical features typical of hydrogels, which have a continuous liquid water phase. To differentiate these edible oil gels from conventional organo-gels, which are typically organic solvent gels used in various industrial applications in the chemical sector, they are now referred to as oleogels [31]. 

### 2.2. Oleogelators: Properties and Classification

The conversion of the liquid oil into a solid structure is ensured by the oleogelator, which is responsible for forming the three-dimensional network in which the oil is trapped. The oleogelation process and the properties of oleogels depend mainly on the oleogelator, but these cannot be discussed without also considering the oil characteristics and the processing conditions [27,48]. Regardless of the oleogelator’s type, when used in food processing, it must possess certain properties including food grade, GRAS status (Generally Recognized as Safe), no negative effects on sensory attributes of the food, similar characteristics to solid fats, stability and, last but not least, economic feasibility [29,49].

In recent years, a large number of oleogelators have been investigated with promising results. These can be classified according to two major criteria (i) gelation strategy on the one hand [50] and (ii) their molecular weight on the other [51]. 

The gelation strategy depends on the solubility of the oleogelator. Thus, lipophilic gelators (e.g., fatty acids, monoglycerides, waxes, phytosterols, ethyl-cellulose) and hydrophilic gelators (generally polymers, including carbohydrates and proteins) can be distinguished [52]. Solubility is an important characteristic of gelators. According to Co and Marangoni [53], there must be a balance between the solubility and insolubility of the gelator. If the solubility is too high, there is a risk of forming a solution instead of a gel, while if the gelator is too insoluble, it will form a precipitate.

**Figure 2:**
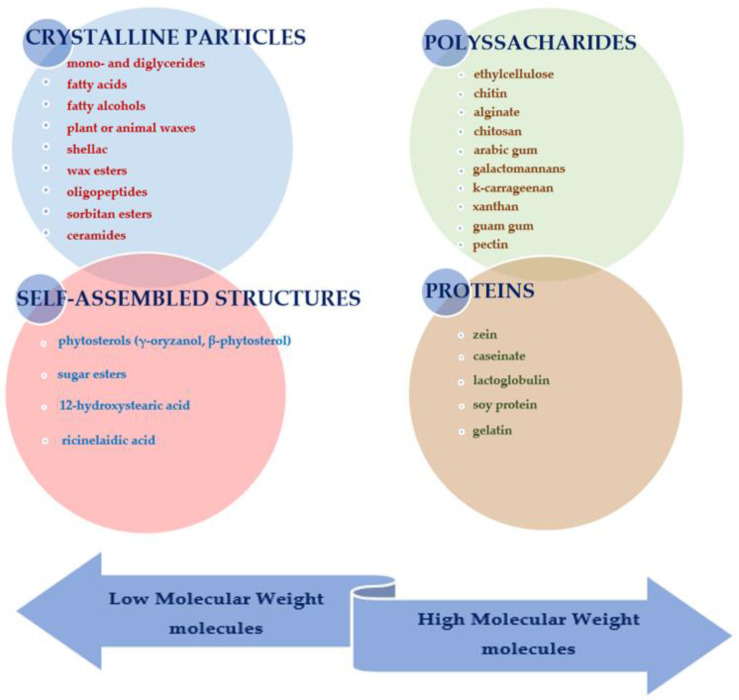
Classification of oleogelators.

Considering their molecular weight, oleogelators can be grouped into low molecular weight molecules and high molecular weight molecules, as shown in Figure 2.

Low molecular weight gelators are small particles with a molecular weight of less than 3000 [54]. According to their gelling behavior, low molecular weight oleogelators can also be subdivided into crystalline particles and self-assembled structures [55].

#### 2.2.1. Crystalline Particles

The most important crystalline particle gelators are mono- and diglycerides, fatty acids and fatty alcohols [56], plant or animal waxes (e.g., rice bran wax, soy wax, sunflower wax, carnauba wax, candelilla wax, beeswax) [30,57], shellac, wax esters, oligopeptides, sorbitan esters (monostearate and tri-stearate), lecithin [58] and ceramides [55]. The gelation mechanism consists of the formation of a crystalline network, by completing three specific stages: nucleation, self-assembly and self-organization. First, the nucleation centers are formed as a result of the precipitation or/and crystallization gelator’s molecules, due to the oversaturation of the solvent [55]. Secondly, through the self-assembly process, the primary particles can grow and form different shapes of crystals (fibers, tubules, rods or ribbons). Finally, the gel is formed as a result of crystalline particles’ self-organization into a continuous network, where the liquid oil is immobilized [29]. The mechanical and rheological properties of the network depend on the crystals’ configuration, which can be controlled through the cooling and shearing processes [59].

#### 2.2.2. Self-Assembled Structures

This group of gelators include phytosterols (γ-oryzanol, β-phytosterol), sugar esters, 12-hydroxystearic acid, and ricinelaidic acid [55]. These compounds are able to form thin fibrils, which intertwine with each other, resulting in a three-dimensional network (structure) sustained by non-covalent bonds, such as hydrogen bonds, Van der Waals forces, *π*-*π* interaction, and electrostatic interactions [36]. 

Proteins and polysaccharides are the two major classes of high molecular weight oil gelators that have attracted attention in the field because they can be used as food ingredients, are widely available, are inexpensive, and some have potential nutritional value [50]. Excepting ethyl-cellulose and chitin [60], the high molecular weight oleogelators are difficult to use for structuring hydrophobic oils due to their hydrophilic nature. Thus, their usage requires a different technology, as described in the following paragraph. Polysaccharides such as alginate, chitosan, Arabic gum, galactomannans [59], k-carrageenan [61], xanthan and Guam gum [62], pectin [63] and proteins such as zein [64], caseinate, lactoglobulin, soy protein and gelatin [13] are the most commonly used high molecular weight compounds used in oleogelation.

### 2.3. Methods of Obtaining Oleogels 

Depending on the solubility of the oleogelator, oleogels can be obtained by two distinct methods: direct or indirect dispersion. An important aspect in this regard is that neither of these methods produces changes in the chemical or structural properties of the oils so that their nutritional value is not affected [59].

#### 2.3.1. Direct Dispersion

Direct dispersion, due to its simplicity, is one of the most widely used methods of producing oleogels. The method is specific for hydrophobic agents and consists of direct dispersion of the oleogelator into the oil, heated to a temperature above its melting point. Then, to achieve the gelation, the mixture is cooled, at which point the nucleation and crystal growth leads to the formation of the gelator network and the entrapment of the oil in this solid structure [65]. 

In terms of texture, stability, rheological or thermal properties, oleogels’ characteristics might be improved by using mixtures of two or even more oleogelators with synergistic interaction. Some of the most representative oleogelators’ systems include stearyl alcohol and stearic acid, stearyl alcohol, stearic acid and ethyl-cellulose [66], monoglycerides and phytosterols [67], adipic acid and ethyl-cellulose [68], candelilla wax and glyceryl monostearate [69], tripalmitin with candelilla wax, lecithin and fruit wax [70], γ-oryzanol and β-sitosterol [71], lecithin and stearic acid [72]. Table 1 summarizes some notable examples found in the scientific literature regarding the procedure for the direct dispersion method for obtaining oleogels.

#### 2.3.2. Indirect Dispersion

Indirect methods of obtaining structured oils have attracted considerable attention in recent years because they can diversify the range of oleogelators, to include also hydrophilic compounds, such as proteins and polysaccharides, which cannot be directly dispersed in oil. Indirect dispersion involves the formation of a structural network or building blocks in a water-continuous system, followed by the removal of the aqueous phase, as shown in Table 2. This can be achieved by various techniques, such as the biphasic emulsion method, solvent exchange, or foaming method [29,30].

(i) Biphasic emulsion method

As described by Alvarez et al. [88] and Li et al. [26], this method first involves the preparation of a concentrated oil in water emulsion, by using a protein as an emulsifier. Then, the emulsion is dried and sheared, resulting in stable oleogels with a hydrophilic gelator and a high concentration of edible oil (above 97%).

(ii) Solvent exchange

The solvent exchange method consists primarily of forming a hydrocolloid by dispersing a polymeric material in water, followed by heating to expose its hydrophobic groups. This results in a strong network due to the physical cohesive forces, ionic or covalent bonds between the polymeric chains of proteins or polysaccharides. Next, water is removed and replaced by an organic solvent with medium polarity (acetone, tetrahydrofuran) [89] so as to prevent any disruption of the network by the solvent exchange procedure. The solvent is then substituted with oil, leading to the oleogel formation [60,90].

(iii) Foam-templated method

The foam-templated method is based on the preparation of aqueous protein foams, followed by the removal of water through drying, which leads to an aerogel structure. The dried foam is immersed in oil until saturation and sheared to break the polymeric structure and to obtain oleogels [13]. Sometimes, for obtaining stable foams, polysaccharides such as pectin, alginate, carrageenan [50], xanthan gum and Tara gum [91] can be added.

**Table 2:** Examples of oleogels obtained by indirect methods.

Oleagelator	Oil	Gelation Conditions	Application	Reference
Hydroxypropyl methylcellulose (HPMC)	Sunflower oil (SFO)	2 wt% aqueous HPMC solution was aerated, frozen at −23 °C, and freeze-dried. The resulting cryogel was submerged in SFO and sheared by centrifugation (11,000 rpm).	Comparative evaluation of different structured oil systems.	[75]
Hydroxypropyl methylcellulose (HPMC) 0.2, 0.4, 0.6, 0.8, 1.0 wt% Xanthan gum (XG) 0.3%	Soybean oil (SO)	SO (60 wt%) was dispersed (13,000 rpm) in an aqueous HPMC solution, followed by adding of XG solution under high-speed shearing. The mixture was dried at 90 °C and resulting product was smashed by a grinder, followed by shearing at 10,000 rpm.	Evaluation and optimization of soybean oil, HPMC and XG oleogel characteristics.	[92]
Gelatin (G) (3%, 5%) Xanthan gum (XG) (0.1%, 0.2%)	Canola oil (CO)	G and XG were dissolved in water, aerated by homogenization (13,000 rpm, 5 min), frozen at −20 °C overnight, and freeze-dried (24 h). Cryogel samples were saturated with CO and sheared by homogenization (0.5–2 min, 10,000 rpm).	Study about the ability of G and XG to produce oleogel through foam- templated method.	[93]
Pork skin (PS)	High oleic sunflower oil (HOSO)	PS, cooked for 40 min at 80 °C and comminuted in a blender, water and HOSO were mixed in the ratio of 1.5:1.5:1.	Replacement of 50% pork backfat in bologna sausages.	[94]
Canola protein isolate (CPI)	Canola oil (CO)	50% CO in water emulsion stabilized by high-pressure homogenization with 4% CPI was heated at 90 °C for 30 min and dried at a 0.4 atm vacuum and 60 °C, followed by shearing.	Replacing 50% of traditional shortening in the cake batter.	[95]
Xanthan gum (XG) 0.3 wt% Hydroxypropyl methylcellulose (HPMC) 0.6 wt%	Soybean oil (SO)	60% SO was dispersed (13,000 rpm) in HPMC aqueous solution and mixed with XG solution under high-speed shearing. The mixture was dried (90 °C) and smashed by a grinder (10,000 rpm).	Characterization of structure and molecular properties of polysaccharide oleogels.	[92]
Methylcellulose (MC) Hydroxypropyl methylcellulose (HPMC)	Sunflower oil (SFO)	Emulsion template approach (ETA): MC/HPMC aqueous solution (1.5 *w*/*w*%) and SFO (18%, 33%, 47% *w*/*w*) mixture was homogenized with water (16,500 rpm/1 min). The emulsion was dried (60 °C, 48 h) and sheared. Foam template approach (FTA): MC/HPMC aqueous solution (2 *w*/*w*%) was homogenized (16,500 rpm/2 min) and lyophilized. The sample was minced and saturated with SFO.	Comparison between ETA and FTA for designing edible oleogels based on cellulose ethers.	[96]
Regenerated keratin (RCh)	Sunflower oil (SFO)	2 g of SFO was added to 6.5 g of RCh aqueous suspension (0.4–1.4 %) and emulsified by ultrasonication (2 min, 60% sonication amplitude). The emulsion was freeze-dried and sheared.	Characterization of SFO/RCh oleogel in terms of morphology, thermal behavior and viscoelastic properties.	[97])
Citrus pectin (CP) Tea polyphenol-palmitate (TP)	Camellia oil (CO)	TP (2.5%) was melted in CO and cooled, followed by dispersion of CP (1.5–4.5% *m/v*) at room temperature. The mixture was emulsified by high-speed shearing (20,000 rpm/2 min), freeze-dried (48 h) and sheared (10,000 rpm/2 min).	Characterization of CO oleogel structured with TP and CP.	[63]

### 2.4. Advantages and Benefits of the Use of Oleogels in Foods 

The major health benefit that may be achieved by using oleogels is the substitution of saturated and trans fats with unsaturated fats. Through the process of oleogelation, it is possible to produce a managed or controlled distribution of functional ingredients and medicines that have been added to meals in the appropriate quantities [90]. 

The human body absorbs, in very small proportions, several fat-soluble compounds, including lycopene, coenzyme Q10, β-carotene, conjugated linoleic acid, eicosapentaenoic acid, plant sterols, isoflavone, docosahexaenoic acid and tannins [98]. Their ingestion results in medical or health advantages, including the prevention and treatment of certain illnesses, e.g., reduction in platelet aggregation, blood viscosity and fibrinogen, as well as antioxidant qualities and a reduced prevalence of chronic diseases such as cardiovascular diseases and different types of cancer. For this reason, their encapsulation or delivery via oleogels is needed, by including the latter in functional foods [99].

Numerous experimental results obtained by scientists who formulated meat products containing oleogels may be a starting point for the meat industry to implement this alternative formulation and for nutritional and technological considerations. It is generally accepted that hard=stock is a natural source of trans-fats, and it has been claimed that processed meat products contain an average of 35% saturated fats [99,100]. This is one of the primary factors that contribute to the prevalence of cardiovascular diseases among consumers [100].

Fats are responsible for both the structure and flavor of meat products. The composition of fats has binding capabilities, which further contribute to the consistency and durability of meat products. As customers’ tastes are also influenced by sensorial characteristics, innovative formulations need to preserve these qualities which have traditionally been associated with meat products [90,101]. 

In addition to being used in the production of foods that can withstand high temperatures, oleogels can also be used to solve the problem of oil leaks in a broad variety of different products. Oleogels also have significant secondary utility as transporters for lipid-soluble bioactive chemicals, which is another important application. Recent uses of oleogels have reduced the possibility of trans-fatty acids being created during the frying of noodles, although the frying of foods in oil at a high temperature is a substantial contributor to the synthesis of trans-fatty acids [102].

In the chocolate industry, oleogels are mainly used to solve one of the most pressing problems of replacing oil binders in chocolate paste, to prevent fat bloom during storage and to make chocolate more heat-resistant. To combat this issue in hot climates, chocolates can be formulated with added high-melting oleogels, making them thermally resistant. Saturated fatty acids were reduced by 30% due to the 27% rapeseed oil instead of palm oil. During storage, no oil separation occurred [103].

Ethyl-cellulose was the oleogelator of corn-oil based oleogel shown to increase the hardness of dark chocolate when used to replace 50% of the cocoa butter, while glycerol monostearate was the only oleogelator of the named oleogel that formed solid like chocolate by replacing 100% of the cocoa butter. Based on these results, oleogels rich in unsaturated fatty acids may be useful agents for reducing cacao butter in dark chocolate [104]. 

Few experiments have attempted to replace milk fat with oleogel in dairy products. Oleogel, a milk fat alternative, has been extensively studied as a possible addition to cream cheese products. Different types of cream cheese, including those produced with rice bran wax and ethyl=cellulose oleogels, have been developed and compared to both regular and reduced-fat commercial cream cheese as standards. The use of oleogel improved the fatty acid profile of the cream cheese, as total fat levels were reduced by 25% in all samples compared to the full-fat commercial control [105].

In a separate study, researchers analyzed the use of oleogels instead of milk cream in artisanal ice cream. The oleogel-enriched samples showed a more optimal ratio of healthy to less healthy fatty acids. For instance, sunflower oil-based oleogel containing 12 g/100 g gelators produced ice creams with the same or even better-quality characteristics than versions produced with milk cream. Therefore, using oleogels in ice creams as milk cream substitutes have proven to be a practical way to achieve healthier products [106]. 

For baked products, such as cookies and bread, saturated fat is a necessary ingredient to maintain their specific taste, elasticity and texture. By replacing regular margarine with oleogels produced from edible oils and natural waxes, it is possible to produce cookies with less saturated fat. Oleogels are a lower calorie and more environmentally friendly substitute for saturated and hydrogenated fats in bakery products, without lowering the overall standard of the end product. Researchers working in the field of healthy food design are paying close attention to the many applications that structured oils can have in the baking industry [107,108]. 

Saturated and trans fats are often ingested through margarine consumption. As solid substitutes for margarine, it is possible to use oleogels, which offer superior texture, color and oxidation stability during storage. Several variants of oleogels have been studied, but those based on vegetable oils rich in polyunsaturated fatty acids using natural waxes as oleogelators have shown good results in obtaining healthy margarine and spreads. However, consumers prefer to consume commercial margarine instead of that prepared with wax-based oleogel because of its less waxy aftertaste. It is possible to obtain spreadable commercial margarine with a candelilla wax-beeswax content of less than 3%. In addition, combining two different waxes will make the oleogel-based margarine harder, while the melting point can be adjusted by changing the ratio of the different waxes used [109].

## 3. Trends in Improving Nutritional Profile of Meat Products by Using Oleogels

Meat and meat products are an important part of people’s diets everywhere, as they contain significant amounts of proteins and have a high nutritional value. However, due to their high content of saturated and trans fatty acids, consumption of these foods can be a health hazard. A high intake of saturated fats correlates with an increased risk of several diseases, such as diabetes, obesity, inflammation, high blood lipid levels, oxidative stress, metabolic syndrome and, in particular, cardiovascular diseases. The meat industry is therefore obliged to develop healthier meat product alternatives with an improved fatty acid profile to protect the health of customers. Technological improvement of the fatty acid profile can be achieved by replacing animal fat, which meat products naturally contain, with fats that have a more favorable profile [110].

Today’s consumers are more health-conscious than ever and are therefore willing to pay extra for healthier meat products. In this sense, the first step could be the elimination of fat from processed meat products, but fat plays a crucial role in the ability of a meat product to retain its sensory, technical, textural, oxidative stabilization and flavor compound-producing capabilities. Changes in fat content and composition can have adverse effects on the fundamental qualities of products, reducing their attractiveness to consumers and reducing their marketability. Therefore, fat replacers should be nutritionally superior to animal fat, but retain the same structure and other quality attributes as real fat [90]. 

In terms of their composition, fats and oils are made up of triacyl-glycerides, molecules formed by binding three fatty acids to glycerol. For this reason, the quality of the fat is determined by its types of included fatty acids, namely saturated fatty acids, -*cis* and -*trans* monounsaturated fatty acids and -*cis* polyunsaturated fatty acids. When the saturation level is high, more solids are produced and these solids can self-assemble into crystalline structures formed from triacylglycerols. The temperature may also be responsible for inducing this final form, which results in a more extensive crystal network. Solid fats contribute to the texture and stability, as well as the function of the product. Therefore, possible fat replacers should offer results not only in terms of nutritional content but also in terms of solid structure and proper flavor [2]. 

Designing alternatives to animal fat requires a deep analysis of the qualities of the lipid material, which in turn dictate the characteristics of the product into which it will be incorporated. Meat fat has a wide variety of quality characteristics such as appearance, texture, resistance to lipid oxidation, flavor and many others. The use of a lower-quality animal fat substitute in meat products can cause many technological problems, such as improper drying, oily appearance, rancidity and others. It is therefore very important to consider the quality requirements for new fat materials, such as their fatty acid content and physical qualities [2].

**Figure 3:**
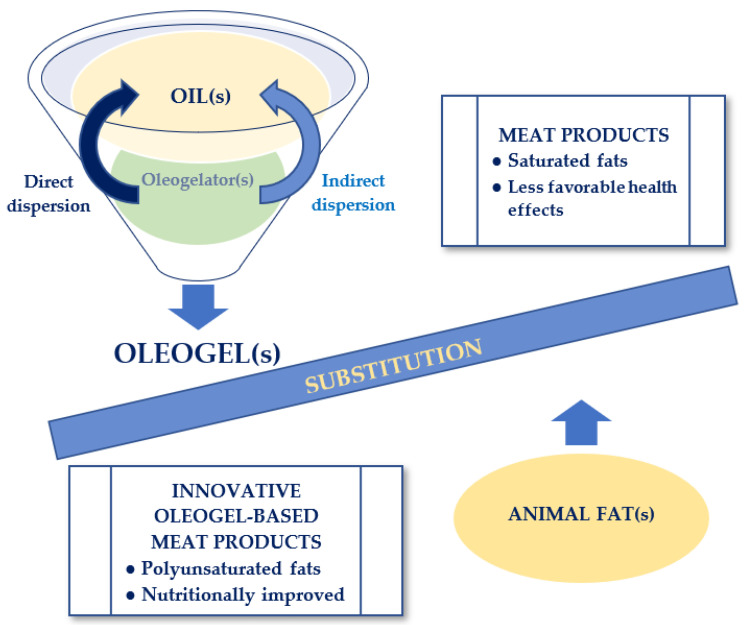
Viable alternative solution for animal fat substitution in meat products.

A possible alternative solution for replacing animal fats in meat products, intensively researched in recent years as a response to consumers’ health needs, is presented in Figure 3. 

Efforts have been made to replace meat fat with liquid oils. The first mention of oleogels in meat products explains that the creation of extremely small fat globules is one of the key problems of direct oil-enrichment, along with high vulnerability to oxidative breakdown and low solubility [2].

Oleogelation, which allows the addition of vegetable oil, is one of the most promising methods for structuring by using oleogelators that include crystalline particles, fatty acids, monoacylglycerols and waxes, supramolecular structures and the polymeric molecule ethyl cellulose. The selection of the vegetable oil to be used in the development of oleogels is important not only in terms of its nutritional properties but also in terms of its fatty acid composition, which influences several characteristics of oleogels, such as crystallization and melting behavior, rheological properties and oxidative stability [101]. 

Oleogelation is a method of stabilizing and adjusting the consistency of food suspensions and edible oils. The use of this technology has shown encouraging results in addressing the physical and sensory difficulties in meat products, which are related to the direct replacement of saturated and trans fats with vegetable oils. The most common types of oil used in processing meat products include sunflower, sesame, soybean, olive, fish and even linseed oil [29]. 

In terms of oleogelators used to structure edible oils, it is a common practice to use monoglycerides to generate economically viable oleogels. In the process of making healthier frankfurters, half of the pig backfat was replaced by sunflower oil-based oleogel, gelled with a combination of 1:1 monoglycerides and 3:1 phytosterols. The resulting frankfurter acquired higher hardness values compared to the control. In addition, oxidation degree and sensory parameters were not altered due to this replacement [67]. 

There is a wide variety of wax oleogelators available, some of them from plant provenance, e.g., candelilla, carnauba, rice bran and sunflower, and others from animals, such as beeswax. Relative to commercial meat products, it has been shown that the aroma and taste of waxes produce the least possible sensory changes in meat products formulated with wax-based oleogels [111]. 

Due to their ability to give meat products a crispy and elastic appearance and to serve as a stabilizer and glaze component, it is feasible to use waxes as oleogelators. Beeswax, at a concentration of 11%, added to a mixture of linseed, fish and olive oils, produced a high oleic acid oleogel, further used to create low-fat pork burgers enriched in polyunsaturated fatty acids. To reduce lipid oxidation during refrigeration and cooking procedures, curcumin was added at a rate of 0.2% [2,111,112]. In another study, linseed oil was gelled with 8% beeswax and the obtained oleogel was used to formulate frankfurters, as a substitute for 25% and 50% of pork fat. The low *n*-6/*n*-3 resulting ratio was a strong indicator of the increased frankfurters’ nutritional value. In addition, fat replacement showed minor effects on textural qualities, although improvements in sensory characteristics are still needed for consumer acceptance [90]. Beeswax has also been used in high-fat meat products (liver pâté), with positive results in the case of a mixture of vegetable oils (olive, linseed and fish) [111].

Phytosterols are an interesting group of oleogelators for the production of oleogels due to their structure, which is very similar to that of cholesterol in human cells. It has also been shown that phytosterols can reduce the amount of cholesterol in human blood by up to 10%. Even though a combination of ɣ-oryzanol and β-sitosterol in linseed oil is a nutritionally favorable choice, there are additional technical considerations that need to be considered. Linseed oil gelled with this sterol combination, at a concentration of 8%, has been shown to be particularly effective in replacing 25–75% of the fatty acids in pork meatballs. For the same reason, pork meatballs have been formulated with the addition of *Fucus vesiculosus* extract (250–1000 ppm) to prevent oxidation of the linseed oil during storage [67,113]. 

By plasticizing the ethyl cellulose structure, ethyl cellulose oleogels can take on the melting and flow characteristics of various lipids. The addition of adipic acid to ethyl cellulose improved the flexibility of oleogels when used in meat products. By formulating beef burgers with 2% ethyl cellulose and 4% adipic acid oleogel, a pleasing textural profile, color and organoleptic qualities were noticed [68]. 

## 4. Designing of Innovative Oleogel-Based Meat Products

The stability of a meat emulsion depends on several factors, such as the size of the fat globules (smaller globules are more stable), the degree of breakdown of the cell structure to release the fat and the amount of dissolved protein covering the contact area of the fat droplets. The fat content of processed meat products varies between about 20% and 30% and has significant interactions with the other elements of the emulsion. Fat plays a key role in the quality of meat products, as it gives the proper tenderness, moisture, taste and appearance. It is a challenge for meat processors to create low-fat meat products, both healthier and with satisfying sensorial properties. When solid fats are replaced by liquid oils, the fatty acid profile of meat products improves, as this increases the amount of mono and polyunsaturated fatty acids and improves the ratio of n-6 to n-3 acids [114]. 

For this purpose, it is essential to discover economically feasible procedures that can lower the amount of fat considered to be included in meat products. In this sense, several recent studies regarding oleogels’ use in various meat products as animal fat replacers are briefly presented in Table 3.

The information presented in Table 3 proves that the substitution of the animal fat with differently designed oleogels and their incorporation in meat products resulted in better fatty acid profiles and more acceptable sensory and technical properties.

## 5. Conclusions and Future Perspectives

In concordance with the recent trends in attention to human health, many researchers have shown the possibility of totally or partially replacing animal fat in meat products. Without minimizing the solution of incorporated cereal flours, fibers, natural bio-polymers, or plant-based emulsions, notable results were obtained by using oleogels.

Oleogel production and integration into foods presents a growing interest among researchers, constituting an important advance in food technology. This is due to their ease of obtaining, affordability and versatility. The understanding of oleogels’ characteristics and their capacity of controlling phase separation and of decreasing the mobility of the oil phase is extremely important for developing novel applications. There is still a real need to investigate different formulations of oleogels from the point of view of component type and ratio, as well as properties that make them suitable for meat products. The influence of different processing parameters on the oleogels’ properties also needs to be examined further. Despite the promising results of obtaining healthier meat products with a higher fatty acid profile, some negative aspects, in terms of textural parameters, flavor or oxidative stability, have also been reported, thus further studies are needed to eliminate these drawbacks. However, promoting the benefits of meat products reformulation with oleogels to producers is highly recommended and can be considered a future direction in obtaining healthier foods.

## Figures and Tables

**Table 1:** Examples of oleogels obtained by direct dispersion method.

Oleagelator	Oil	Gelation Conditions	Application	Reference
Candelilla wax (CW) 3 and 6%	Canola oil (CO)	Heating to 150 °C, under gentle agitation for 15 min and cooling at room temperature.	Partial replacement of shortening in cookies.	[73]
Sunflower and Carnauba waxes (SW, CW) 3, 7 and 10%	Hazelnut oil (HO)	Heating to 90 °C, stirring vigorously for 5 min and cooling at ambient temperature.	Comparative study with a commercial shortening.	[74]
Shellac wax (SW) 0–6 wt%	Rapeseed oil (RO)	The oil and wax dispersion was heated at 90 °C, under mild agitation for 30 min.	Comparative evaluation of different structured oil systems.	[75]
Candelilla wax (CW) 10%	Walnut oil (WO)	Dispersion of oleogelator in the oil, followed by heating at the melting point of oleogelator under stirring (450 rot/min) and cooling down at 0–4 °C.	Nutritional improvement of chocolate spread.	[76]
Glyceryl monostearate (GMS) 10%				
Soy wax (SW) 11% Soy lecithin (SYL) 5 mg/30.6 g	Refined soybean oil (RSO)	Melting SW in RSO, at 65 °C under stirring (300 rpm) for 15 min, followed by cooling at 4 °C for 90 min.	Optimization of RSO-SW oleogel characteristics by adding SYL.	[77]
β-sitosterol / stearic acid mass ratio: 1:0, 4:1, 3:2, 2:3, 1:4, 0:1	Sunflower oil (SFO)	The gelator/oil mixtures (20 g/100 g) were heated at 90 °C under stirring (400 rpm) and cooled at ambient temperature.	Evaluation and optimization of the characteristics of sunflower oil, β-sitosterol and stearic acid oleogel.	[78]
Glycerol monolaurate (GML) 1, 3, 5 and 10 wt%	Camellia oil (CO)	Camellia oil (CO) and GML mixtures were constantly stirred at 80 °C and cooled at room temperature.	Improving physical properties and oxidation stability of Camellia oil (CO) by oleogelation.	[79]
Candelilla wax (CDW) and glyceryl monostearate (GMS) mixture 80:20, 60:40, 40:60, 20:80 (*w*/*w*)	Canola oil (CO)	Melting CDW and GMS mixture in CO at 90 °C under stirring at a weight ratio of 9:1, until the complete dissolution, followed by cooling at 25 °C for 24 h.	Improving physicochemical properties of oleogels with binary blends of oleogelators.	[80]
Carnauba wax (CW)/adipic acid (AA) mixture 50:10, 40:20, 30:30, 20:40, 10:50 (*w*/*w*)	Soybean oil (SO)	CW/AA (6%) were dissolved in SO at 150 °C until complete dissolution and cooled down at ambient temperature (1 °C/min).	Carnauba wax/adipic acid oleogel characterization for fat replacement in cake and beef burger.	[81]
Ethyl-cellulose (EC) 5 wt%; 10 wt%	Corn oil (CO)	EC powder was mixed with heated CO (150 °C), stirred (15 min) and cooled at room temperature.	Oleogel rheological and tribological properties evaluation.	[82]
Sunflower wax (SW) (5%) sorbitan monostearate (SP); stearyl alcohol (SA) (0.05% *w*/*w*)	Sunflower oil (SFO)	Direct dispersion of SP and SA in heated mixture (80 °C) of SW and SFO, followed by cooling at room temperature.	Improving oleogels physicochemical properties by addition of SP and SA emulsifiers.	[56]
Beeswax (BW) 4, 5, 6, 8% *w*/*w* Carnauba wax (CW) 4, 5, 6, 8% *w*/*w*	Pumpkin seed oil (PSO)	Direct dispersion of oleogelators in heated oil (80 °C for BW and 90 °C for CW) under stirring (200 rpm), followed by cooling at 25 °C.	Physical characterization of pumpkin seed oil oleogel.	[83]
Behenyl alcohol (BO) and behenic acid (BA) mixture, in different weight ratio	Sunflower oil (SFO)	Dispersion of BO and BA mixture (10%) in a heated SO (85 °C) until complete dissolution and cooling at room temperature.	Improving oleogel properties by mixing BO and BA oleogelators.	[84]
β-sitosterol, γ-oryzanol (3:2 *w*/*w* mixture)	Flax-seed oil (FSO), sunflower oil (SFO), olive oil (OO), triolein, castor oil (CO)	5, 10, 20% (*w*/*w*) oleogelators were mixed with oils, heated at 90 °C under stirring and cooled at 4 °C.	Study on the effect of oil type on the gelation process: gelling time, mechanical and thermal behavior.	[85]
Beeswax (BW) 8%	Linseed oil (LO)	8% (*w*/*w*) BW was dispersed under stirring in heated LO (80 °C) for 30 min and cooled at room temperature.	Fat substitution with LO/BW oleogel in frankfurters.	[86]
Ethyl-cellulose (EC) (0–10% *w*/*w*)	Soybean oil (SO)	EC was added to the heated SO (140 °C) under stirring (14 min), followed by cooling at room temperature.	Characterization of thermo-oxidative behavior of EC oleogels.	[87]

**Table 3:** Overview of some recent studies on the integration of oleogels in meat products.

Meat Product	Oleogel Components and Preparation		Level of Animal Fat Substitution	Effects of Oleogel Incorporation on the Formulated Meat Product	Reference
	Vegetable Oil(s)	Oleogelator(s)			
Beef burgers	Sesame oil (SO)	Beeswax (BW)	25% and 50% by animal fat with 10% BW oleogel	Decrease of more than 50% in the hardness, chewiness, gumminess, and lightness of raw burgers. Reduction of 11% in cooking loss and 1.6% in fat absorption after processing; color insignificantly changed; lipid oxidation significantly increased. Cooked burgers could not mimic the control in terms of textural properties, but proved good acceptability.	[115]
	5%, 7.5%, 10% BW (*w*/*w*), dissolved in SO under vacuum at 70 °C				
Beef burgers	Soybean oil (SO)	Carnauba wax (CW) and	50% by bovine fat	Acceptable texture profile, color and organoleptic characteristics, comparable with the un-substituted product.	[81]
	2% CW reinforced with 4% AA for improving the thermal behaviour and crystallinity, dissolved in SO at 150 °C				
Burgers	Olive oil (OO)	Pork skin (PS)	100% by bovine backfat	Reduction of 80% in fat content, 35% decrease in energy value, 15% higher protein content and a better fatty acid profile. After processing at 180 °C, the hardness and chewiness, sensory characteristics and overall acceptability proved high and comparable to the control. Better oxidative stability than the control over 7 days at 4 °C.	[116]
	PS in coarse powder form:deionized water:OO 1:3:1, mixed under heating at 100 °C				
Pork burgers	Mixture of olive oil (44.39%), linseed oil (37.87%) and fish oil (17.74%)	Ethyl cellulose (EC) Beeswax (BW)	6% by pork backfat	Acceptable technological properties and good sensory acceptability. Decrease of lipid oxidation during storage or cooking due to the addition of curcumin, but reduced sensory acceptance. Regarding the use of EC in the present combination, further studies are needed to reduce lipid oxidation during refrigeration and cooking and to increase the sensory acceptability of burgers.	[117]
	11% EC or 11% BW, incorporated in the oil mixture while adding or unadding 0.2% curcumin as antioxidant				
Cooked meat batters	Soybean oil (SO)	Mixture of Ethyl cellulose, Avicel RC-591 and α-cellulose 67.0:16.5:16.5	100% by pork backfat	Considerably improved polyunsaturated fatty acids profile of meat batters, decreased lipid oxidation, with unaffected texture and acceptance. Darker and less red color than the control, but more yellow due to the presence of SO.	[118]
	11% celluloses mixture and 3.67% of Span^®^ 60 as surfactant (*w*/*w*), dissolved in 85.33% SO at 120 °C				
Meat patties	Canola oil (CO)	Hydroxypropyl methylcellulose (HPMC)	50% and 100% by beef tallow	Enhanced quality attributes, such as lower cooking loss, softer texture and reduced fatty acids levels from 42% to 15%, by replacing 50% of the beef tallow; lowered saturated to unsaturated fat ratio, from 0.73 to 0.18.	[119]
	2, 4, 6% (*w*/*w*) of 1% HPMC aqueous solution in the form of freeze-dried and grinded foam, added into CO and sheared				
Beef heart patties	Rapeseed oil (RO)	Beeswax (BW)	100% by beef fat	Improved nutritional, fatty acid profile and cooking loss by incorporating the 10% BW oleogel; decreased hardness and oxidative stability during cold storage.	[120]
	2.5, 5, 7.5, 10, 12.5% BW (*w*/*w*), dissolved in RO at 90 °C				
Meat-based pâté	Linseed oil (LO)	Beeswax (BW)	30% and 60% by pork subcutaneous fat	Improved nutritional value by the increased polyunsaturated fatty acids content and decreased omega-6/omega-3 ratio up to 90%. Decreased hardness and adhesivity.	[121]
	8% BW (*w*/*w*), dispersed in LO at 80 °C				
Frankfurters	Soybean oil (SO)	Rice bran wax (RBW)	100% by pork backfat	Acceptable technological quality in terms of emulsion stability, cook/chill yields and oxidation stability. No substantial differences for adhesiveness, cohesiveness, and chewiness in relation to the control. Significantly reduced flavor.	[122]
	2.5% and 10% RBW (*w*/*w*), mixed with SO at 90 °C				
Chicken-based bologna sausages	High-oleic oil (HOSO) and conventional soybean oil (CSO)	Rice bran wax (RBW)	100% by pork backfat	Similar quality and organoleptic properties between formulated sausages, when used HOSO and CSO oleogels. Higher nutritional value when used HOSO oleogel.	[123]
	10% and 2.5% RBW, mixed with 90% and 97.5% HOSO or CSO at 90 °C				
Bologna sausages	Conventional sunflower oil (SFO) or high oleic sunflower oil (HOSO)	Glyceryl monostearate (GM)	25, 50, 75 and 100% by pork fat	Stable emulsions and good sensory acceptance of the sausages, with no significant differences between treatments. Higher level of unsaturated fatty acids and a more compact structure that affected the sliceability. Reducing the pork fat by 50% proved to be the best option, which not affected the hardness of the sausages.	[124]
	5% GM (*w*/*w*), mixed with SFO or HOSO at 90 °C				
Thai sweet sausages	Rice bran oil (RBO)	Rice bran wax (RBW)	25%, 50%, and 75% by total fat	Reduction in total content of saturated fat and cholesterol, but increased softness of sausages. Replacing the 50% of fat with RBW oleogel showed the highest score of overall acceptance.	[125]
	RBW and RBO, mixed at the ratio 2:100 g (*w*/*w*) at 80 °C				
Fermented sausages	Linseed oil (LO)	Mixture of γ-oryzanol and β-sitosterol (60:40 *w*/*w*) Beeswax (BW)	20% and 40% by pork backfat	Substantial quality changes in terms of pH, color, texture. Improvement of polyunsaturated fatty acid/saturated fatty acid and n-6/n-3 ratios. The textural parameters of formulated sausages need to be improved for a better acceptability.	[126]
	8% oleogelators (*w*/*w*), dissolved in LO at 80 °C				
Fermented pork sausages	Olive oil (OO)	Monoglycerides (MG)	50% by pork backfat (in addition, 50% of NaCl was replaced by KCl)	Significant changes in the physicochemical and microbiological properties of the sausages. Acceptable sensory attributes and a healthier nutritional profile than the control. Additional studies are needed for improving the sensory characteristics and consumer acceptability.	[47]
	15% MG, dissolved in OO at 90–95 °C				
Frankfurters	Canola oil (CO)	Ethyl cellulose (EC)	Substitution of beef fat so as to obtain 18.2% fat provided by oleogel in meat batters	Lower sensory hardness when adding 1.5% or 3.0% SMS than the sample with 0.0% SMS, at the low EC levels. Similarity between the oleogel sample with 8% EC and the control and significantly increased hardness at higher EC concentrations. The acceptance of the product unaltered by the replacements.	[127]
	8, 10, 12, 14% EC; 8, 10, 12, 14% EC and 1.5% SMS; 8, 10, 12, 14% EC and 3.0% SMS, mixed with CO at 140 °C				
Breakfast sausages	Canola oil (CO)	Ethyl cellulose (EC)	Substitution of pork fat so as to obtain 20.8% fat provided by oleogel in sausages. 8% rusk was added	Similar objective hardness of the most SMS oleogels compared with the pork fat control sample. No rancidity and chemical taste for the final product, prevented by the addition of BHT and rosemary oleoresin to CO. No changes in the water and fat contents during the heating of sausages, due to the presence of the rusk that contributed to their binding.	[128]
	8, 10, 12, 14% EC and 1.5, 3.0% SMS, mixed with CO added with 50 ppm BHT and 0.6% rosemary oleoresin, for preventing heat-induced oxidation of the oil, at 170 °C				
Frankfurters	Canola Oil (CO)	Ethyl cellulose (EC)	20%, 40%, 60%, and 80% by beef fat	Diminished hardness of the frankfurters compared to the control, but similar shear forces for all samples. No differences detected by sensory analysis compared to the control. Decreased water loss and smokehouse yield.	[129]
	8% EC and 1.5% SMS; 8% EC and 3.0% SMS; 10% EC and 1.5% SMS, mixed with CO at 170 °C				
Sucuk	Flaxseed oil (FO)	Sunflower wax (SFW) Beeswax (BW)	Oleogels were included at 17.17% in the same recipe as the control containing tallow fat	Significantly higher concentrations of unsaturated fatty acids content, but lower texture and sensory attributes than the control, such as hardness, chewiness, juiciness, fattiness, aroma, and flavor.	[130]
	10% SFW or BW (*w*/*w*), dissolved in FO at 80 °C				
Liver pâté	Canola oil (CO)	Ethyl cellulose (EC) and Glycerol monostearate (GMS)	20%, 40%, 60%, 80%, 100% by lard	The hardness, oiliness, cohesiveness, and perceived off-flavors undifferentiated in relation to the control for all samples. The replacement of 60% lard is recommended, in terms of oil retention, proper texture, color, and due to the reduced saturated fat content of about 40%.	[131]
	12% EC and 3% GMS, mixed with CO at 140 °C				
Pâtés	Mixture of olive (44.39%), linseed (37.87%), and fish (17.74%) oils	Ethyl cellulose (EC) Beeswax (BW)	Partial or total substitution of pork backfat so as to obtain 15% fat content pâtés	Optimal fatty acid profile (high PUFA/SFA and low n-6/n-3 ratios), but a significantly increased lipid oxidation. Insignificantly affected emulsion stability, color and texture, compared to the control. The sensory characteristics not significantly affected by using BW oleogel, but a negative effect on sensorial properties when using EC oleogel, in direct relation with the substitution level.	[111]
	11% EC and 3.67% SMS, mixed with oils at 160 °C 11% BW, mixed with oils at 65 °C				

Note: The controls refer to the samples without oleogel addition. All mixtures were obtained under stirring. Abbreviations: SMS—Sorbitan monostearate, AA—adipic acid, BHT—butylated hydroxyl toluene.

## Data Availability

Not applicable.
